# Integrative analysis of the late maturation programme and desiccation tolerance mechanisms in intermediate coffee seeds

**DOI:** 10.1093/jxb/erx492

**Published:** 2018-01-29

**Authors:** Stéphane Dussert, Julien Serret, Aldecinei Bastos-Siqueira, Fabienne Morcillo, Eveline Déchamp, Valérie Rofidal, Philippe Lashermes, Hervé Etienne, Thierry JOët

**Affiliations:** 1 IRD, Université Montpellier, UMR DIADE, France; 2 CIRAD, Université Montpellier, UMR DIADE, France; 3 CIRAD, Université Montpellier, UMR IPME, France; 4 Biochimie et physiologie moléculaire des plantes, CNRS, INRA, Montpellier Supagro, Université Montpellier, France

**Keywords:** *Agrobacterium* transformation, *Coffea arabica*, desiccation tolerance, heat-stable proteome, intermediate seed, late-embryogenesis abundant protein, late maturation, heat-shock factor, heat-shock protein, transcriptome

## Abstract

The ‘intermediate seed’ category was defined in the early 1990s using coffee (*Coffea arabica*) as a model. In contrast to orthodox seeds, intermediate seeds cannot survive complete drying, which is a major constraint for seed storage and has implications for both biodiversity conservation and agricultural purposes. However, intermediate seeds are considerably more tolerant to drying than recalcitrant seeds, which are highly sensitive to desiccation. To gain insight into the mechanisms governing such differences, changes in desiccation tolerance (DT), hormone contents, and the transcriptome were analysed in developing coffee seeds. Acquisition of DT coincided with a dramatic transcriptional switch characterised by the repression of primary metabolism, photosynthesis, and respiration, and the up-regulation of genes coding for late-embryogenesis abundant (LEA) proteins, heat-shock proteins (HSPs), and antioxidant enzymes. Analysis of the heat-stable proteome in mature coffee seeds confirmed the accumulation of LEA proteins identified at the transcript level. Transcriptome analysis also suggested a major role for ABA and for the transcription factors CaHSFA9, CaDREB2G, CaANAC029, CaPLATZ, and CaDOG-like in DT acquisition. The ability of CaHSFA9 and CaDREB2G to trigger HSP gene transcription was validated by *Agrobacterium*-mediated transformation of coffee somatic embryos.

## Teaser

Integrative analysis of developing coffee seeds reveals the key cellular, metabolic, and regulatory processes associated with acquisition of desiccation tolerance in intermediate seeds

## Introduction

The seed is a key structure in the life cycle of higher plants; it facilitates dispersal in space and over time, and consequently ensures the survival of the species. It has been proposed that the majority of flowering plants produce seeds that are able to withstand almost complete loss of cellular water. Together with certain desiccation-tolerant invertebrate animals, such as bdelloid rotifers, tardigrades, and nematodes, resurrection plants, many yeast cells, and fungal spores, most plant seeds are anhydrobiotic organisms (Crowe *et al.*, 1992). In the dry quiescent state, these seeds are endowed with an exceptional capacity to endure extreme conditions after dispersal, such as intense frost/heat events or long drought periods (Buitink and Leprince, 2008). In addition, the longevity of such seeds increases in a predictable way by reducing seed moisture content and temperature (Roberts, 1973), and these so-called ‘orthodox’ seeds can therefore survive *ex situ* storage for very long periods under conventional gene-bank conditions (Li and Pritchard, 2009).

However, about 8% of the world’s flowering plants produce seeds that do not tolerate desiccation (Tweddle *et al.*, 2003; Wyse and Dickie, 2017). Two other categories of seeds – intermediate and recalcitrant – have thus been defined with respect to their storability in gene banks (Roberts, 1973; Ellis *et al.*, 1990). These terms are now commonly used to describe the level of sensitivity to drying of non-orthodox seeds. Although the level of desiccation tolerance (DT) may vary considerably within each of these two seed categories (Dussert *et al.*, 1999; Berjak and Pammenter, 2008), it is commonly acknowledged that recalcitrant seeds do not survive if dried to water potentials of about –15 MPa, equivalent to about 90% relative humidity (RH), while intermediate seeds are able to withstand drying to about 30% RH (Black and Pritchard, 2002). Plants that produce non-orthodox seeds prevail in specific habitats, such as tropical rain forests where they represent >45% of tree species (Tweddle *et al.*, 2003; Hamilton *et al.*, 2013). Seeds of many major tropical crops are recalcitrant (e.g. cocoa, coconut, rubber tree) or intermediate (e.g. tea, coffee, oil palm, citrus), which represents a major constraint for growers, seed companies, and germplasm repositories (Li and Pritchard, 2009; Walters *et al.*, 2013).

At least four types of mechanisms, which act synergically and appear to be shared by all anhydrobiotic organisms, mitigate the deleterious effects of desiccation (Crowe *et al.*, 1992; Hoekstra *et al.*, 2001; Leprince and Buitink, 2010; Gaff and Oliver, 2013; Tapia and Koshland, 2014): (i) prevention of oxidative damage through accumulation of antioxidant compounds, such as tocols and glutathione, together with co-ordinated repression of basal metabolism during drying; (ii) avoidance of structural stress through cell wall modification, reorganisation of intracellular membranes and cytoskeleton, and chromatin condensation; (iii) stabilisation of membranes and proteins by non-reducing sugars, late-embryogenesis abundant proteins (LEA) and heat-shock proteins (HSP) through the formation of hydrogen bonds with polar residues of proteins and membrane phospholipids, which maintain dry molecules in a physical state similar to that seen in the presence of water (a mechanism known as the ‘water replacement hypothesis’, reviewed in Crowe, 2007); and (iv) efficient detoxication and repair systems for damaged DNA and proteins upon rehydration. Several recent comparative transcriptomic, proteomic, or metabolomic studies have determined how these mechanisms are orchestrated during dehydration and rehydration in anhydrobiotes, such as resurrection plants, bryophytes, tardigrades, and midges (Oliver *et al.*, 2011; Yobi *et al.*, 2012; Dinakar and Bartels, 2013; Gusev *et al.*, 2014; Wang *et al.*, 2014).

In orthodox seeds, DT is acquired *in planta* during the late maturation stage of seed development. Again, proteome, transcriptome, and gene co-expression network analyses have greatly improved our understanding of DT acquisition in orthodox seeds (Buitink *et al.*, 2006; Chatelain *et al.*, 2012; Verdier *et al.*, 2013). In maturing orthodox seeds, the down-regulation of genes involved in the cell cycle, DNA processing, and primary metabolism, and the up-regulation of genes coding for small HSPs (sHSPs) and LEAs, and of genes involved in stress defence (antioxidant system, secondary metabolites) coincide with DT acquisition (Buitink *et al.*, 2006). The specific role of certain LEA proteins in DT has been further characterised through proteome analysis of developing *Medicago truncatula* seeds (Chatelain *et al.*, 2012) and of pre-germinated *M. truncatula* and *Arabidopsis thaliana* seeds treated with polyethylene glycol to re-induce DT, which was lost during germination (Boudet *et al.*, 2006; Maia *et al.*, 2011).

The co-ordinated induction of these protective mechanisms involves complex regulatory networks and signalling pathways (Verdier *et al.*, 2013). In *A. thaliana*, seed maturation is controlled by several master regulators, which interact in a complex manner linked with ABA signalling (Karssen *et al.*, 1983; Gutierrez *et al.*, 2007). They include the CCAAT-box binding factor LEC1 and the three B3 domain-containing proteins ABI3, FUS3, and LEC2 (To *et al.*, 2006). Their downstream targets include other transcription factors (TFs), hormonal pathways, and LEA genes (To *et al.*, 2006; Santos-Mendoza *et al.*, 2008). Part of the seed maturation process is therefore indirectly controlled by master regulators via secondary TFs, which activate their own transcriptional programme (Mönke *et al.*, 2012). Such a cascade has been described between ABI3 and Heat Shock Factor A9 (HSFA9), a secondary TF responsible for HSP transcriptional activation (Kotak *et al.*, 2007). Analysis of gene co-expression networks during *M. truncatula* seed development also revealed close associations between ABI3, secondary TFs such as ABI4, ABI5, AP2/EREBP, and HSFs, and many LEA genes (Verdier *et al.*, 2013; Zinsmeister *et al.*, 2016).

In contrast to the considerable progress made in understanding DT acquisition during the development of orthodox seeds, our knowledge of the factors involved in desiccation sensitivity of intermediate and recalcitrant seeds remains very limited. Reactive oxygen species (ROS) are directly implicated in recalcitrant seed death following partial dehydration (Leprince *et al.*, 1999; Bailly, 2004; Berjak and Pammenter, 2008). The lack of co-ordinated repression of metabolism during drying, e.g. unabated respiratory activity and subsequent metabolic dysfunction, is thought to play a predominant role in the oxidative burst that triggers lipid oxidation, membrane disruption, and ultimately the death of recalcitrant seeds. Comparative analyses with orthodox seeds has also revealed quantitative differences in key protective components such as LEAs (Delahaie *et al.*, 2013) and the lack of structural adaptation to prevent mechanical damage (Berjak and Pammenter, 2008).

The basis of intermediate seed sensitivity to desiccation is even less well documented than that of recalcitrant seeds. The features that intermediate seeds would need to be as tolerant to desiccation as orthodox seeds and those that make them considerably less sensitive to drying than recalcitrant seeds are totally unknown. The intermediate seed category has been defined using coffee (*Coffea arabica*) as a model (Ellis *et al.*, 1990). Coffee seeds die when dried below 20% RH (Dussert *et al.*, 2006). The coffee seed is composed of a copious living endosperm surrounding a tiny spatulated embryo (1% of the mature seed mass) (Eira *et al.*, 2006). The embryo is more tolerant to drying than the endosperm and the level of desiccation sensitivity of whole mature seeds actually corresponds to that of the endosperm (Dussert *et al.*, 2006). Species of the genus *Coffea* vary considerably in the level of desiccation sensitivity of their seeds (Dussert *et al.*, 2000), but neither the total lipid and fatty acid content of the endosperm (Dussert *et al.*, 2001) nor the content of non-reducing sugars (Chabrillange *et al.*, 2000) explain the interspecific variation in seed DT. Since these early studies, no progress has been made in identifying the reasons for coffee seed sensitivity to drying, whereas their developmental features and the transcriptional programme for the accumulation of reserves are now better understood (Joët *et al.*, 2009, 2010, 2014). In the present study, changes in transcript abundance and hormone contents of developing coffee seeds were investigated to better understand DT acquisition in intermediate seeds. The roles of two TFs in sHSP accumulation were validated by *Agrobacterium*-mediated transformation of coffee somatic embryos, and changes in LEA transcript accumulation were further characterised by analysing the heat-stable proteome of mature seeds.

## Material and methods

### Plant material, desiccation tolerance, and viability assays

Experiments were performed with seeds of *Coffea arabica* cv. ‘Laurina’. Developing seeds were harvested from plants grown at Grand Tampon, Reunion Island (1015 m above sea level) with an average temperature of 18.5 °C and mean annual rainfall of 1300 mm. For transcriptome and hormone analyses, three independent biological samples (pools of ~200 seeds) were collected from 20 trees randomly selected in the plot for each of the seven developmental stages studied (Salmona *et al.*, 2008). After being cross-sectioned, the seed was separated from the pericarp and immediately frozen in liquid nitrogen and stored at –80 °C. For germination capacity and DT assays, one pool of 300 seeds was collected at 10-d intervals from 140 to 260 d after flowering (DAF), as described above. For DT measurement, batches of 50 seeds were desiccated for 20 d at 25 °C in the dark over saturated solutions of KOH (9% RH), K acetate (23% RH), MgCl_2_ (32% RH), K_2_CO_3_ (45% RH), NH_4_NO_3_ (62% RH), or (NH_4_)_2_SO_4_ (81% RH). Seed moisture content, seed germination and viability, zygotic embryo extraction, *in vitro* culture and viability were then assessed as described in Dussert *et al.* (2006). For DT measurement, somatic embryos (SE) were desiccated for 6 h at 25 °C in the dark over saturated solutions of (NH_4_)_2_SO_4_ (81% RH), KCl (85% RH), KNO_3_ (92% RH), or K_2_SO_4_ (97% RH). SE viability was assessed using the criterion of normal autotrophic seedling conversion after 8 weeks of *in vitro* culture at 25 °C in the dark on the maturation medium M described by Etienne (2005).

### Hormone analysis

Seed tissues were freeze-dried and ground to fine powder in an analytical grinder (IKA A10, Staufen, Germany) before being sent to the National Research Council, Canada for hormone analysis (50 mg of dry powder per sample). ABA and ABA metabolites, cytokinins, auxins, and GAs were quantified by ultra-performance liquid chromatography-ESI-tandem mass spectrometry as described in detail by Chiwocha *et al.* (2003). The values presented are means of triplicate determinations.

### Transcriptome analysis

Gene identifiers are from the Coffee Genome Hub (coffee-genome.org). For microarray analyses, total RNA was extracted from whole seeds as described in Joët *et al.* (2014). However, since the embryo represents less than 1% of total seed mass, it was assumed that its contribution to the total RNA population was negligible. Microarrays were performed at the MGX transcriptomic platform (Montpellier-GenomiX, Institut de Génomique Fonctionnelle, Montpellier) using the 15k PUCE CAFE and the protocols described in Privat *et al.* (2011). The microarray data are publicly available at http://www.ncbi.nlm.nih.gov/geo/ (GEO accession number GSE107949). For 454 pyrosequencing, total RNA was extracted from separated endosperm and embryos using the RNeasy™ Lipid Tissue Kit (Qiagen). cDNAs were tagged independently (Titanium Kit, Roche Diagnostics) then mixed for sequencing by GATC Biotech AG (Germany) using GS FLX Titanium (Roche). Raw data have been deposited at the European Nucleotide Archive (ENA) under the project number PRJEB23959. Trimmed reads were mapped on CDS using the BWA-MEM package with default parameters (Li, 2013). Samtools (Li *et al.*, 2009) was used to count mapped reads and the number of reads per kilobase and million reads (rpkm) were then calculated. Statistical analyses of microarray and 454 data were performed as described in Dussert *et al.* (2013) and Joët *et al.* (2014). Briefly, differences in transcript accumulation between two consecutive stages were tested using the Limma Package (Wettenhall and Smyth, 2004) and Audic-Claverie statistics (Audic and Claverie, 1997), respectively. In both cases, *P*-values were Bonferroni-corrected to control for false discovery rates. Hierarchical cluster analysis (HCA) was used to group genes according to their transcription profile using the tool developed by Eisen *et al.* (1998). Gene ontology (GO) annotation was performed using Blast2GO software (Götz *et al.*, 2008) with default parameters. Fisher’s exact test was then used to evaluate the significance of GO term enrichment. Biological enrichment analysis was also performed using Arabidopsis homologues of differentially expressed coffee genes and the Classification Superviewer tool (http://bar.utoronto.ca;Provart *et al.*, 2003) for Mapman categorisation. The method used for qPCR analysis is described in Joët *et al.* (2014). The set of primers that enabled amplification of target genes is detailed in Supplementary Table 1Supplementary Table S1 at *JXB* online. The level of expression of each gene was normalised to the geometric mean of expression levels of three validated reference coffee genes (Cc08_g05690 Ubiquitin UBQ10, Cc00_g15790 40S ribosomal protein S24, and Cc00_g17460 14-3-3 protein; Cruz *et al.*, 2009; Joët *et al.*, 2014).

### Production of transgenic coffee somatic embryos overexpressing *CaHSFA9* and *CaDREB2* genes

*Coffea arabica* HSFA9 and DREB2 cDNAs (Genbank accession numbers JQ687374 and JQ687375, respectively) were used as a template for PCR amplification using the primers described in Supplementary Table 1Supplementary Table S1 and were cloned into the plant overexpression vector pMDC32. Coffee embryogenic calli (*C. arabica* cv. ‘Caturra’) were genetically transformed using recombinant *Agrobacterium tumefaciens* strain LB1119 containing the recombinant plasmid, as previously described (Ribas *et al.*, 2011). About 10 somatic embryos regenerated from each hygromycin-resistant callus (independent transgenic lines) and four regenerated plantlets were tested for transformation by PCR amplification of the selection gene, *HPTII*. Transgenic lines were then tested for both gene expression and desiccation tolerance.

### Heat-stable proteome analysis

Proteins were extracted from 500 mg of ground coffee endosperm obtained from 25 mature seeds. Heat-stable soluble proteins were extracted according to the method described by Boudet *et al.* (2006) and optimised as described by Chatelain *et al.* (2012). The only modification made in the present work was the addition of dithiothreitol (DTT, 5 mM) and polyvinylpolypyrrolidone (PVPP, 4% w/v) to the extraction buffer to prevent tissue oxidation. Proteome analysis included protein fractionation and tryptic digestion, as described in Decourcelle *et al.* (2015), and LC-MS/MS analysis using the mass spectrometer QExactive Plus (ThermoFisher Scientific Inc) (see Supplementary Table 1Supplementary Table S2). Raw files were analysed using Mascot (2.4.0) with a threshold *P*-value of 0.05 and the predicted peptide database from the Coffee Genome Hub.

## Results

### Acquisition of desiccation tolerance, hormonal changes, and transcriptional switches in the developing and maturing coffee seed

**Fig. 1.**
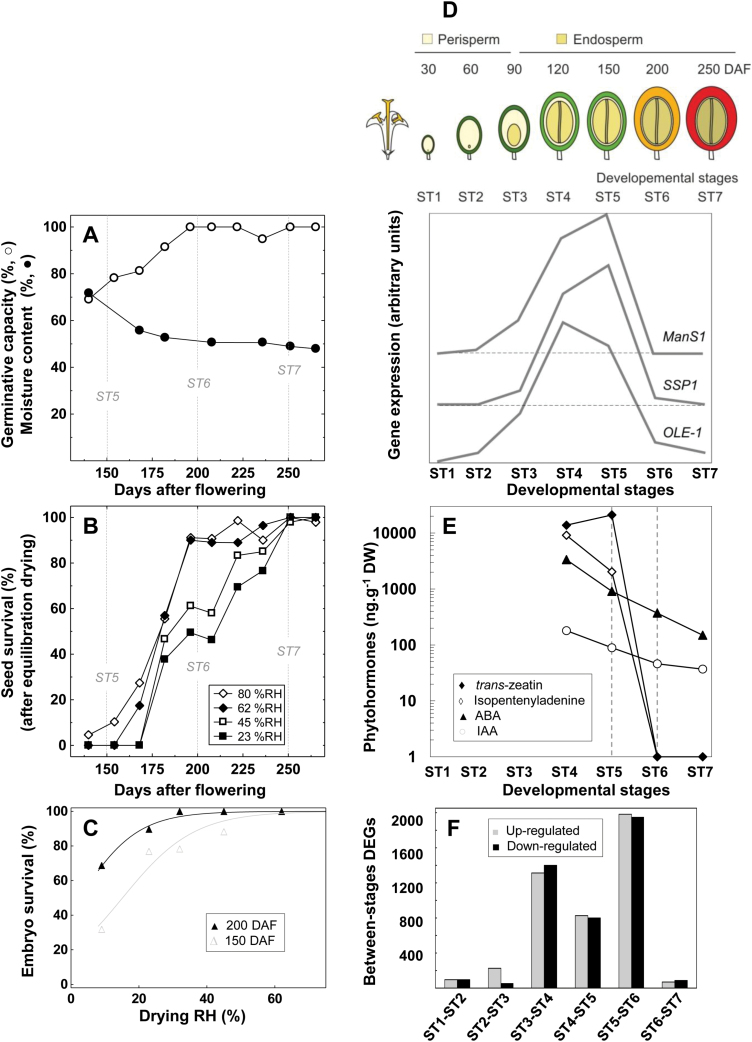
Acquisition of desiccation tolerance (DT) during coffee seed development. (A) Acquisition of germinative capacity and changes in moisture content (on a fresh weight basis) during coffee seed maturation. (B) Changes in viability (%) after equilibration drying at 81%, 62%, 45%, and 23% relative humidity (RH) during seed development. (C) Viability of zygotic embryos at 150 d after flowering (DAF, stage 5) and 200 DAF (stage 6) after equilibration drying at 9%, 23%, 32%, 45% and 62% RH. (D) Schematic representation of the seven developmental stages studied and transcript profile of three genes chosen to illustrate accumulation of storage proteins, triacylglycerols, and cell wall polysaccharides: 11S globulin (*SSP1*, Cc03_g05570), oleosin (*OLE-1*, Cc02_g04750), and mannan synthase (*ManS1*, Cc06_g04240), respectively. (E) Changes in ABA, cytokinins (isopentenyladenine, *trans*-Zeatin), and auxin (IAA) during seed maturation. The DT acquisition timeframe, i.e. the stage 5–6 transition, is indicated by the dashed vertical lines. (F) Numbers of up- and down-regulated genes at each of the six developmental transitions.

Under the field conditions used in this study, seeds completed their development in approximately 260 d. At 150 DAF, most seeds were already able to germinate and to develop into normal seedlings (Fig. 1A) but they could not withstand drying in any of the four RH tested (Fig. 1B). In contrast, at this stage, 75% of embryos extracted from seeds dried in 23% RH germinated normally (Fig. 1C), demonstrating that desiccation-induced loss of viability of immature seeds was only due to damage to the endosperm. Between 150 and 190 DAF, almost all seeds acquired the capacity to be dried in 62% RH and half of them also became tolerant to drying in 23% RH (Fig. 1B). The level of DT of mature coffee seeds was thus mostly gained between 150 and 190 DAF, which corresponds to the transition between stages 5 and 6 according to the anatomic and metabolic criteria defined in previous studies, in which seven stages are distinguished during seed development (Salmona *et al.*, 2008; Joët *et al.*, 2009). These seven developmental stages can be briefly described as follows (Fig. 1D): from stage 1 to stage 2, the perisperm undergoes significant growth, which determines the final size of the seed; at stage 3, the endosperm develops rapidly and replaces the perisperm in the locule; endosperm growth ends by stage 4 and oil starts to accumulate; stage 5 is characterised by endosperm hardening due to the massive deposition of galactomannans in cell walls; accumulation of reserves ends by stage 6, as illustrated by the transcription pattern of three genes representative of oil (*OLE-1*), protein (*SSP1*), and galactomannan (*ManS1*) storage (Fig. 1D), when the pericarp of the fruit turns yellow; finally, fruit and seed maturity is completed at stage 7, when the pericarp becomes red.

In addition, DT acquisition was concomitant with a slight decrease in seed water content (Fig. 1A), most likely caused by the end of reserve accumulation (Fig. 1D). It did not correspond to a desiccation stage *sensu stricto* since seed water content remained high until maturity, i.e. around 50% on a fresh weight basis (Fig. 1A), neither was DT acquisition coupled with a peak in ABA (Fig. 1E, Supplementary Table 1Supplementary Table S3). The highest ABA level was observed at the onset of the accumulation of storage compounds (stage 4) and endosperm ABA content then decreased gradually until maturity. The IAA level showed a similar pattern during endosperm development and maturation. By contrast, a spectacular drop in active cytokinins (trans-zeatin and 2iP) occurred concomitantly with the acquisition of DT between stages 5 and 6, while the level of the inactive glycosylated form of trans-zeatin-O-glucoside remained high until maturity (see Supplementary Table 1Supplementary Table S3). No active gibberellins were detected at any time during endosperm development (Supplementary Table 1Supplementary Table S3). Among the 15 522 genes represented on the PUCE CAFE microarray (Privat *et al.*, 2011), 9573 (61.7%) displayed significant variation in expression during seed development (FDR<0.05; Supplementary Table 1Supplementary Table S4). Among these genes, 5857 were found to be significantly up- or down-regulated between two consecutive stages. This transcriptome survey revealed two major transcriptional switches during seed development (Fig. 1F). The first switch occurred at the transition between stages 3 and 4 (with 1396 and 1312 genes up- and down-regulated, respectively) before the maturation stage (accumulation of reserves). The second major transcriptional switch, of even greater magnitude, occurred between stages 5 and 6 and thus coincided with endosperm DT acquisition and the end of the reserve accumulation programme. About 2000 genes were up-regulated during this stage and approximately the same number were down-regulated (Fig. 1F).

### Acquisition of desiccation tolerance coincided with transcriptional activation of cellular protection mechanisms and co-ordinated repression of metabolic activity

**Fig. 2.**
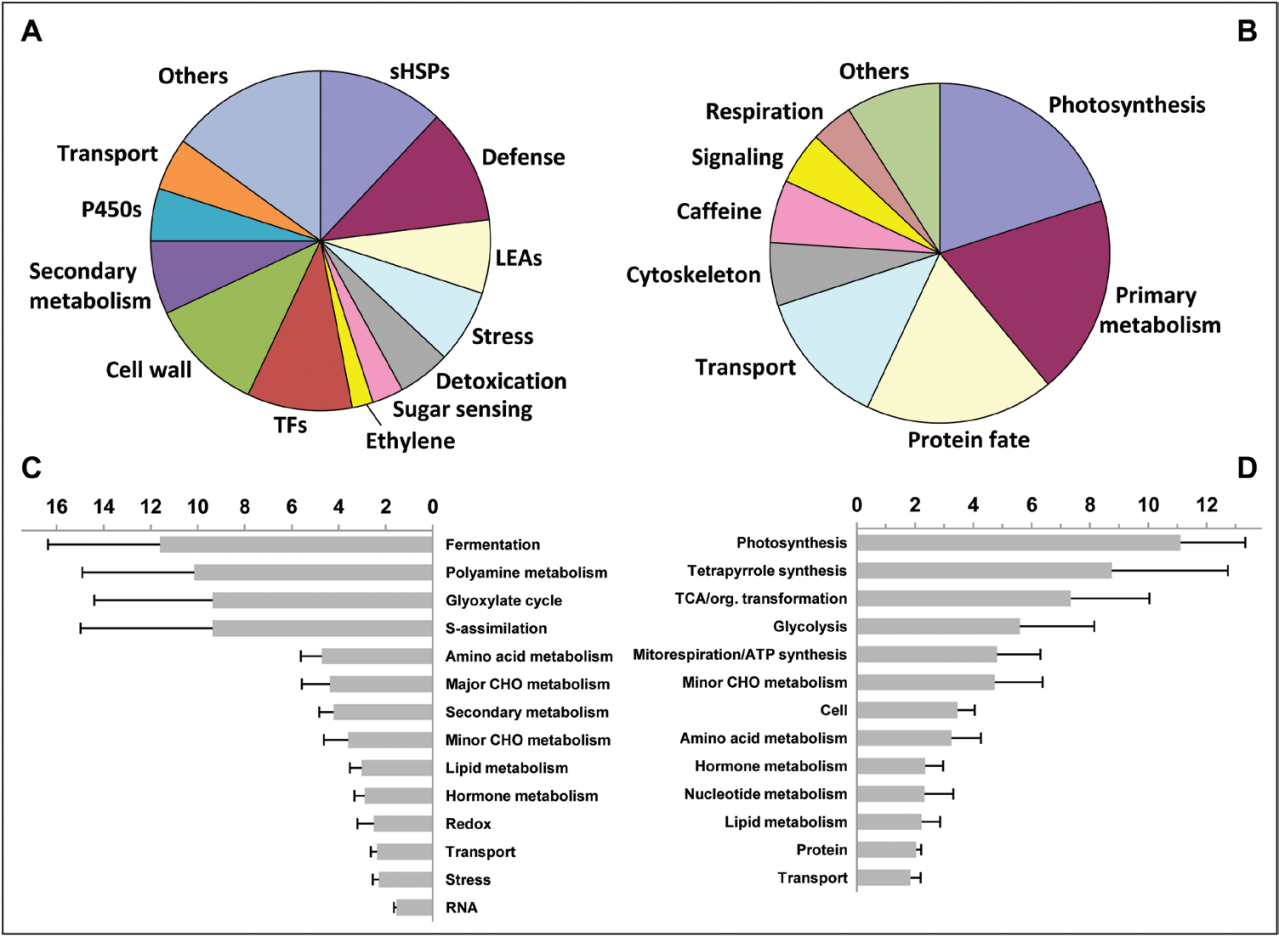
Functional analysis of genes differentially expressed during acquisition of desiccation tolerance (DT) in developing coffee seeds. Pie-charts of functions assigned to the top 100 up-regulated (A) and top 100 down-regulated (B) genes. Functional enrichment analysis (normalised frequencies of Mapman categories and bootstrap standard deviations) of the 1521 up-regulated (C) and the 677 down-regulated genes (D).

Hierarchical clustering analysis (HCA) resulted in 26 major clusters of temporally co-expressed genes (Supplementary Table 1Supplementary Fig. S1). Of these, nine clusters (C18 to C26) grouped 1521 genes specifically up-regulated during DT acquisition (stage 5–6 transition), while cluster C5 contained 613 genes that were specifically down-regulated during this developmental stage (see Supplementary Table 1Supplementary Table S5). All the top 100 up-regulated genes (Fig. 2) displayed a considerable fold-change in gene expression during the stage 5–6 transition: i.e. from 5- to 400-fold changes (Supplementary Table 1Supplementary Table S5). About half of them code for proteins directly involved in cell protection and rescue, including sHSPs, LEAs, defence-, cold-, and drought-induced proteins, ROS-scavenging enzymes, as well as the metabolism of trehalose, a major sugar-sensing and stress-signalling molecule (Fig. 2A). The top 100 up-regulated genes also contained several genes coding for cell wall-modifying enzymes or involved in secondary metabolism, such as genes encoding cytochrome P450 enzymes, and phenylpropanoid and flavonoid biosynthetic enzymes. Finally, transcription regulators represented a substantial proportion of both the top 100 up-regulated genes (10%) and of the full set of genes specifically activated during the stage 5–6 transition (16%; Supplementary Table S6).

The composition of the top 100 down-regulated genes (fold-change between 0.1 and 0.5, Supplementary Table 1Supplementary Table S5) suggests a transcriptional co-ordination of metabolic slowdown during the stage 5–6 transition, with a decrease in the mRNA abundance of genes involved in the fate of proteins (biosynthesis and degradation), primary metabolism, photosynthesis, and respiration (Fig. 2B). The 138 protein-fate genes account for the largest proportion of the transcript repertoire down-regulated during late maturation (32%; Supplementary. Table S6). Genes involved in basal cellular activity, such as those encoding components of cytoskeleton or membrane transporters, were also found to be down-regulated. In addition, functional category enrichment analysis performed on the 424 annotated genes of cluster C5 highlighted global down-regulation of genes involved in energy processes, such as photosynthesis, the tricarboxylic acid cycle, respiratory electron transport, and ATP synthase, and glycolysis (Fig. 2D). Similarly, the most significantly over-represented GO terms in cluster C5 were related to translation, proteasome, photosynthesis, cytoskeleton organisation, and vesicle-mediated transport, while translational activity and organisation of ribosomes were the most significantly under-represented GO terms in up-regulated genes (see Supplementary Table 1Supplementary Table S6).

### Re-routing of energy metabolism and induction of a maturation-specific ROS-scavenging programme

The analysis of up- and down-regulated genes during DT acquisition revealed complex re-routing of energy metabolism, with down-regulation of photosynthesis- and respiration-related transcripts, including key mitorespiration genes, such as those encoding subunits of ATP synthase and electrogenic transporters of the electron transfer chain, and, in counterpart, large transcript accumulation of genes involved in gluconeogenesis, fermentation, glycolysis-related methylglyoxal detoxication, and the glyoxylate cycle (Fig. 2C, Supplementary Table 1Supplementary Table S6). The very few respiratory or photosynthetic genes up-regulated during DT acquisition encode proteins that act as energy-dissipating systems and are involved in the fine-tuning of the redox poise, such as mitochondrial and plastidial alternative terminal oxidases (Cc08_g05330 and Cc09_g01070, respectively), or non-electrogenic (type II) external NADH dehydrogenases (i.e. Cc01_g14360 and Cc02_g12400), which oxidise extra-mitochondrial NADH accumulated in the case of intense glycolytic activity.

**Fig. 3.**
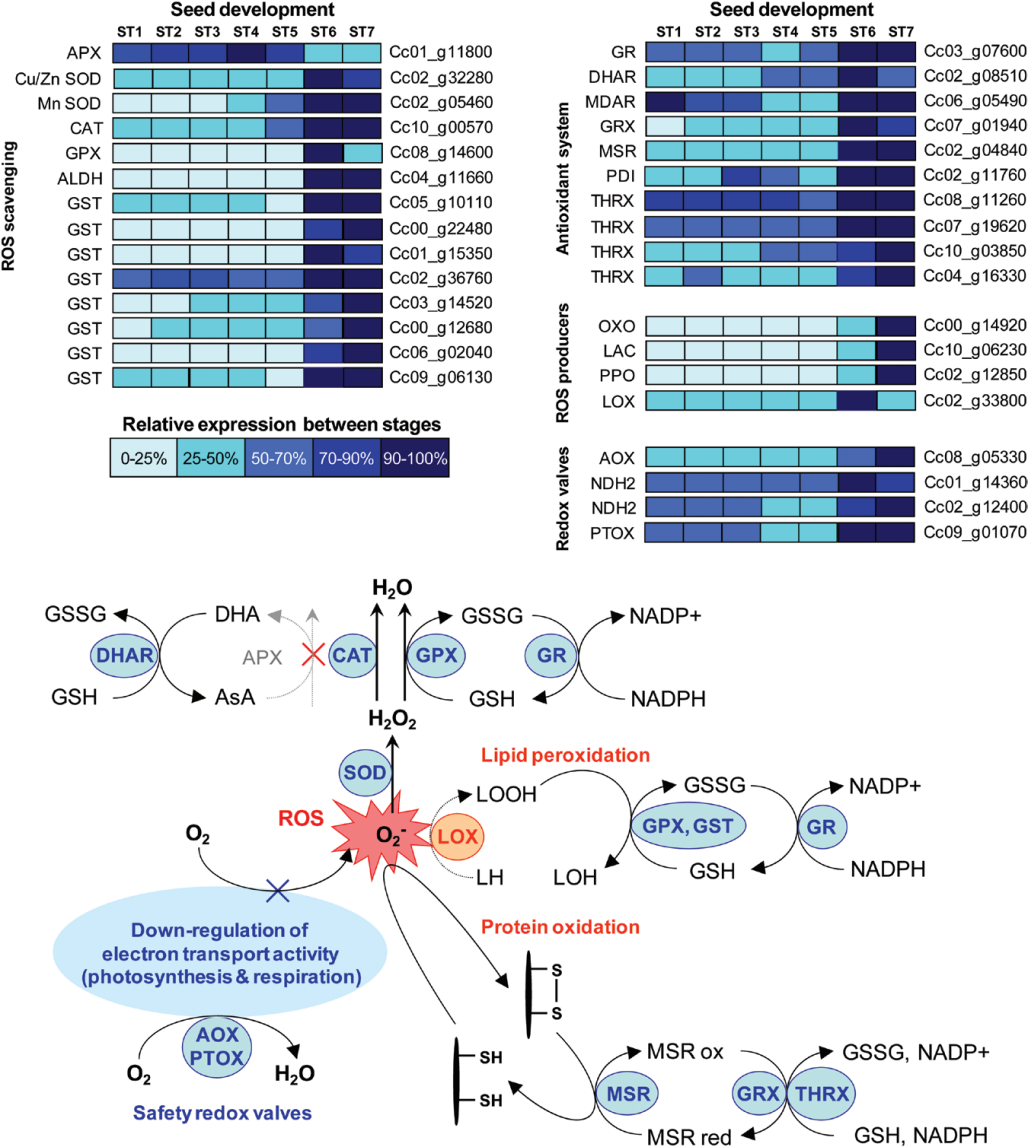
Transcript profiles of desiccation tolerance (DT)-associated reactive oxygen species (ROS) scavenging and detoxification enzymes. Gene expression levels at the seven developmental stages are indicated by the different levels of shading, as indicated in the key, and are presented as percentages of the maximum normalised transcript abundance of the gene. ALDH, aldehyde dehydrogenase; APX, ascorbate peroxidase; AOX, alternative oxidase; CAT, catalase; DHAR, dehydroascorbate reductase; GPX, glutathione peroxidase; GR, glutathione reductase; GRX, glutaredoxin; GST, glutathione-S-transferase; LAC, laccase (diphenol oxidase); LOX, lipoxygenase; MDAR, monodehydroascorbate reductase; MSR, methionine sulfoxide reductase; NDH2, type-2 NAD(P)H dehydrogenase; OXO, oxalate oxidase; PDI, protein disulphide isomerise; PPO, polyphenol oxidase; PTOX, plastid terminal oxidase; SOD, superoxide dismutase; THRX, thioredoxin.

Transcriptome analysis also revealed a major switch in redox homeostasis during the stage 5–6 transition, with the transcriptional activation of many key ROS-scavenging enzymes, such as superoxide dismutases, catalases, glutathione reductases, glutaredoxins, and glutathione peroxidases (Fig. 3). Moreover, many genes encoding the antioxidant and detoxifying enzyme glutathione-S-transferase, which is involved in the reversibility of S-glutathionylation, degradation of xenobiotics, and protein protection against glycation, were also up-regulated during the stage 5–6 transition (Fig. 3). Other genes involved in protection against protein oxidation and glycation, or lipid oxidation, were also highly up-regulated during DT acquisition. These included genes encoding methionine sulfoxide reductase (MSR, Cc02_g04840), glyoxalases, and an aldehyde dehydrogenase (Cc04_g11660) with high homology with Osaldh7, which has been shown to play an important role in seed longevity by detoxifying aldehydes generated by lipid peroxidation (Shin *et al.*, 2009). The induction of a lipocalin gene (Cc06_g15300) homologous to the gene encoding the TIL protein in Arabidopsis, which is involved in seed longevity through lipid protection against oxidation, is also worth noting (Boca *et al.*, 2014). By contrast, an ascorbate peroxidase gene (Cc01_g11800) was down-regulated, suggesting that ascorbate does not play a pivotal role at this stage of seed development.

### Hormone homeostasis and signalling pathways during late maturation

DT acquisition at the stage 5–6 transition coincided with a strong transcriptional activation of several genes primarily involved in the biosynthesis of ABA (zeaxanthin epoxidase, Cc07_g06010, 9-cis-epoxycarotenoid dioxygenase NCED1, Cc05_g10210, xanthoxin dehydrogenase ABA2, Cc01_g08700, and abscisic-aldehyde oxidase, Cc04_g10210), jasmonate (linoleate 9S-lipoxygenase 5, Cc02_g33800), and ethylene (aminocyclopropane-1-carboxylate oxidase ACO1, Cc05_g02890 and Cc09_g01110) (see Supplementary Table 1Supplementary Table S5). However, several genes that control the inactivation and homeostatic regulation of endogenous phytohormone levels were also induced at this stage. Among them, we identified a gene coding for ABA 8′-hydroxylase (Cc08_g05920). The other hormones likely subjected to catabolic or homeostatic processes during DT acquisition were brassinosteroids (cytochrome P450 734A1, Cc04_g03930), gibberellins (gibberellin 2-beta-dioxygenase GA2OX1, Cc05_g10350), jasmonate (amidohydrolase ILL6, Cc10_g12030), cytokinins (zeatin O-glucosyltransferase, ZOG1, Cc06_g17790), and auxin [indole-3-acetic acid (IAA)-amido synthetase GH3.3, Cc02_g19470; UDP-glycosyltransferase 74E2, Cc01_g01490]. In Arabidopsis, the latter two enzymes modulate auxin levels in the presence of excess auxin and under abiotic stress, respectively, while ZOG1 regulates active versus storage forms of cytokinins, and ILL6 contributes to jasmonoyl-isoleucine turnover and jasmonate homeostasis (Staswick *et al.*, 2005, Tognetti *et al.*, 2010, Widemann *et al.*, 2013). Finally, transcript profiles of the cytokinin biosynthetic enzymes adenylate isopentenyltransferase (*IPT1*, Cc11_g10030) and cytokinin riboside 5′-monophosphate phosphoribohydrolase (*LOG1*, Cc06_g03960) mirrored seed cytokinin contents, with high transcript levels at stages 4 and 5 followed by a significant decline in expression at stages 6 and 7.

The repertoire of genes up-regulated during DT acquisition also included proteins involved in intracellular signalling and protein kinase cascades (e.g. MAPK). Among them, we detected several type-2C protein phosphatase genes (PP2Cs; Cc01_g12800, Cc02_g34520, Cc06_g03340), which play important roles in stress signal transduction in plants by negatively regulating the ABA response and MAPK cascade pathways. SRK2I (sucrose non-fermenting 1-related subfamily 2, SnRK2.3, Cc07_g05710), a direct component of the ABA signalling pathway, was also activated during DT acquisition. The induction of the ABA receptor gene *PYR1* (Cc08_g02750) and of the cytokinin receptor *AHK3* (Cc11_g17030), which is a negative regulator of drought and salt stress responses and of ABA signalling (Tran *et al.*, 2007; Kumar and Verslues, 2015), is also worth noting. Several key players of energy homeostasis were also transcriptionally activated, such as trehalose phosphate synthase genes (*TPS*; Cc11_g06690), *KINB1* (Cc01_g06750), and *KING1* (Cc04_g10020, Cc11_g13370), which encode β and γ regulatory subunits of SnRK1, the plant orthologue of the evolutionarily conserved SNF1/AMPK/SnRK1 protein kinase family.

### LEA accumulation in developing coffee seeds

**Fig. 4.**
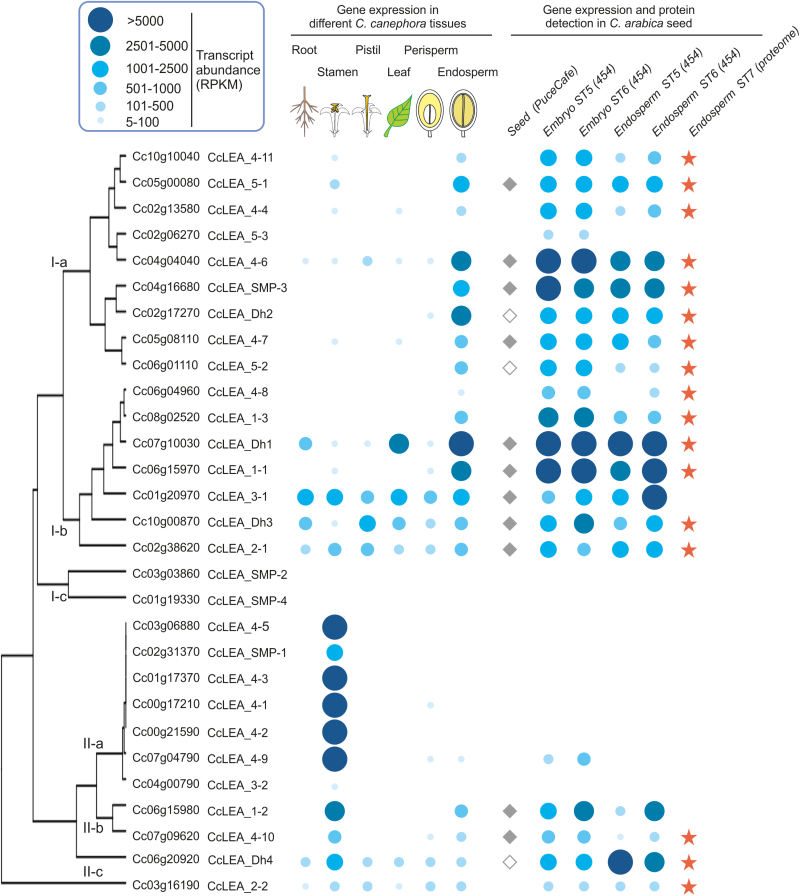
Genome-wide analysis of late-embryogenesis abundant (LEA) proteins in coffee. LEA genes were classified according to hierarchical clustering analysis of transcript abundance in *C. canephora* roots, stamens, pistils, leaves, and perisperm at 180 d after flowering (DAF) and in endosperm at 260 DAF. Expression levels in *C. canephora* tissues and in *C. arabica* zygotic embryos and endosperm at 150 and 200 DAF are indicated by the blue dots, the size and intensity of which represent the number of reads per kilobase and million reads (rpkm; see key). LEA peptides detected in the heat-stable proteome of mature seeds are labelled with an orange star. LEA genes differentially expressed during seed development as shown by microarray data analysis are labelled with a diamond (closed diamonds indicate LEA genes differentially expressed during the stage 5–6 transition).

The amino acid sequences of the 51 Arabidopsis LEAs (Hundertmark and Hincha, 2008) were used to conduct BLAST searches against the coffee genome, which led to the identification of 29 LEA proteins (see Supplementary Table 1Supplementary Table S7). Phylogenetic analysis of all coffee and Arabidopsis LEA proteins (Supplementary Table 1Supplementary Fig. S2) showed that coffee LEAs are distributed in the seven distinct groups defined according to PFAM domains (LEA_1 to LEA_5, dehydrins and seed maturation proteins, SMP; Hundertmark and Hincha, 2008). Using publicly available coffee transcriptomes of various vegetative and reproductive tissues (Denoeud *et al.*, 2014), HCA revealed that only five LEA genes are ubiquitously expressed (*LEA_2-1*, *LEA_2-2*, *LEA_3-1*, *LEA_Dh3*, *LEA_Dh4*; Fig. 4). Most LEAs displayed marked tissue-specificity, with maximum expression in either the stamen (Cluster II-a, seven LEA genes) or the endosperm (Cluster I-a, nine LEA genes). Since not all LEA genes were represented on the PUCE CAFE microarray, we then conducted a 454 pyrosequencing-based transcriptome analysis of the coffee embryo and endosperm at the stage 5–6 transition (Fig. 4, Supplementary Table 1Supplementary Table S8). This analysis showed that 19 LEA genes were very highly expressed in the endosperm at stage 6. Significant amounts of these 19 transcripts were already present at stage 5 in the embryo (150 DAF), in which two additional LEA genes were significantly expressed (*LEA_5-3*, Cc02_g06270 and *LEA_4-9*, Cc07_g04790). LEA genes expressed in the endosperm at stage 6 belonged to all seven groups of LEAs (i.e. three genes in group LEA_1, two in LEA_2, one in LEA_3, six in LEA_4, two in LEA_5, one SMP, and four dehydrins). Within this subset, 13 LEA genes were significantly up-regulated during DT acquisition at the stage 5–6 transition according to Audic-Claverie (454 data) and Limma (microarray) statistics (Supplementary Table 1Supplementary Table S7). To ascertain whether the lower DT of intermediate seeds compared to orthodox seeds was caused by incomplete translation of LEA transcripts during late maturation, we analysed the heat-stable soluble proteome of mature coffee seeds (Supplementary Table 1Supplementary Table S7). Proteins were detected for 17 of the 19 transcripts accumulated at stage 6 (Fig. 4). Except for group LEA_3, all the LEA groups were represented in the mature coffee seed proteome.

### TFs that putatively govern the late maturation programme in coffee seed

Transcriptome profiling revealed intense transcriptional activity during the establishment of DT and, among the up-regulated genes, 128 displayed putative functions related to transcriptional regulation (representing 16% of genes with an assigned putative function; Supplementary Table 1Supplementary Table S5). The TF showing the highest induction was a NAC domain-containing protein 29 (ANAC029, Cc11_g12740) with a 45-fold change during DT acquisition. In addition, the most represented TF classes were AP2/ERF, MYB, WRKY, and HSF families (with more than six genes in each class). Some examples of very highly up-regulated TFs during DT acquisition are homologues of DREB2G (Cc09_g03140, 17-fold change), MYB113 (Cc07_g08030, 15-fold change), HSFA9 (Cc06_g11570, 4-fold change), and four WRKY genes with fold-changes higher than 3 (Cc04_g05080, Cc11_g12480, Cc09_g01430, and Cc01_g14950). Among these candidates, it is worth noting that the *DREB2G* and *HSFA9* genes had large sequence identity with their homologues in Arabidopsis and sunflower (see Supplementary Table 1Supplementary Fig. S3), which have been shown to be involved in drought response and desiccation tolerance (Sakuma *et al.*, 2006; Prieto-Dapena *et al.*, 2006, 2008). Two other TFs with high homology to PLATZ (Cc08_g02880) and Delay of Germination-like (*DOG-like 4*, Cc02_g15140), which are involved in DT acquisition in Arabidopsis seeds (Gonzalez-Morales *et al.*, 2016), were also strongly induced at the stage 5–6 transition (8-fold change for *DOGL4*). Finally, with regards to the master regulators of seed development and maturation, it is worth noting that *ABI3* (Cc01_g17380) and *FUS3* (Cc07_g01190) peaked at stages 4 and 5, respectively, i.e. during seed filling and before DT acquisition (see Supplementary Table 1Supplementary Table S4). Unfortunately, the PUCE CAFE microarray lacks representative probes for coffee genes homologous to *AtABI4* (Cc02_g06230) and *AtABI5* (Cc06_g05680). *CaABI4* was not detected at either stage 5 or 6 in the 454 pyrosequencing-based transcriptome data, and very low transcript amounts were observed for *CaABI5* (Supplementary Table 1Supplementary Table S8).

### Functional validation of HSFA9 and DREB2G

**Fig. 5.**
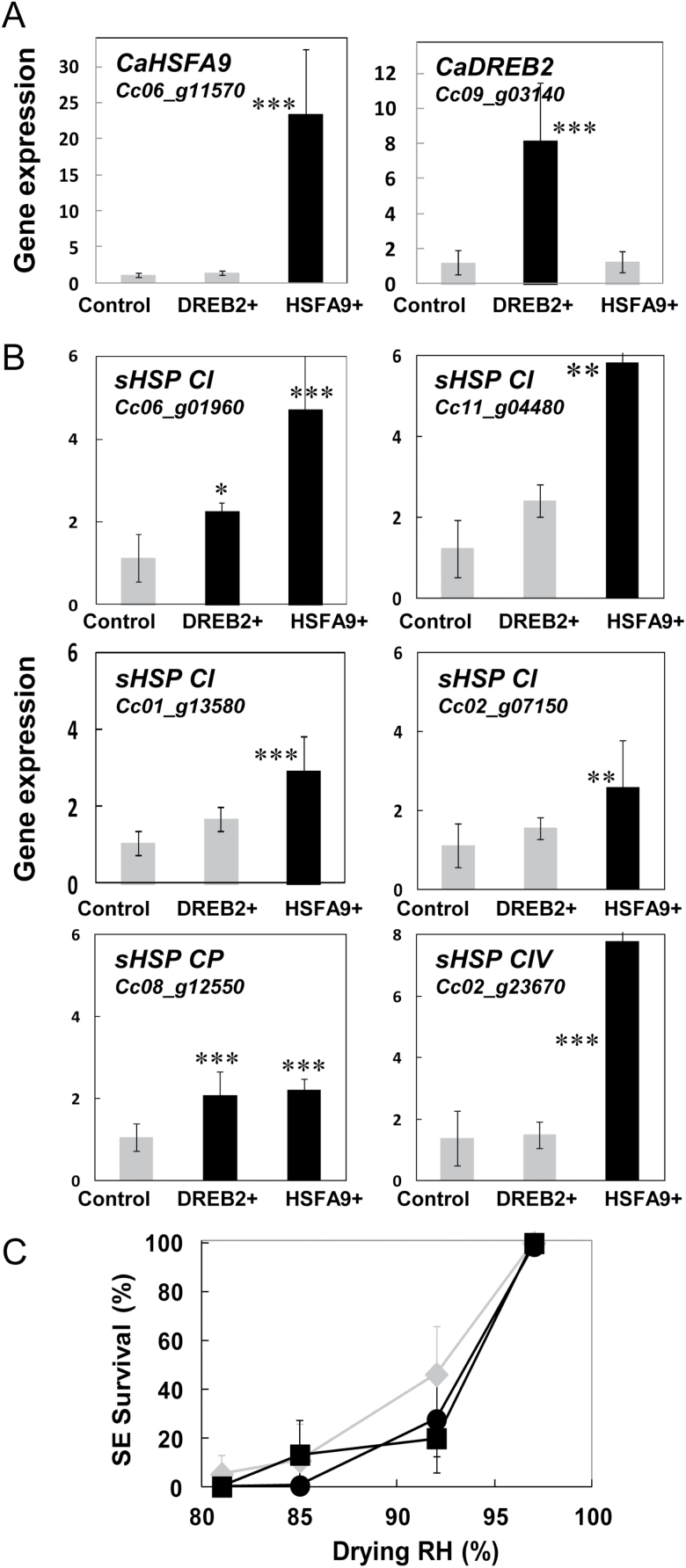
Functional validation of *HSFA9* and *DREB2G.* Small heat-shock protein (sHSP) gene expression and desiccation tolerance. (A) Real-time RT-PCR analysis of *HSFA9* and *DREB2G* ectopic expression in coffee somatic embryos overexpressing *HSFA9* or *DREB2G* (HSFA9+ and DREB2+, respectively). Values are means (±SD) of four independent transgenic lines. (B) Real-time RT-PCR analysis of sHSP gene expression in HSFA9+ and DREB2+ transgenic somatic embryos. (C) Viability of HSFA9+ (circles), DREB2+ (squares), and control (diamonds) somatic coffee embryos after drying to equilibrium at 81%, 85%, 92%, and 97% relative humidity (RH).

Transgenic coffee somatic embryos overexpressing the HSFA9 or the DREB2G cDNA sequences under the control of the doubled constitutive 35S promoter of the Cauliflower mosaic virus were regenerated by *Agrobacterium*-mediated transformation. All the lines tested (four independent lines for each gene) were positively transformed, as shown by the integration of the *hptII* gene (Supplementary Table 1Supplementary Fig. S4). Real-time RT-PCR analysis demonstrated strong expression of the transgenes in the transformed lines. *HSFA9* expression showed a 23-fold increase in HSFA9^+^ overexpressing somatic embryos, while an 8-fold increase was observed for *DREB2G* expression in DREB2G^+^ somatic embryos (Fig. 5A). Transgene functionality was then investigated through the analysis of putative downstream target expression. Six sHSP genes belonging to the top 100 up-regulated genes during DT acquisition were chosen as putative targets. All six sHSP genes were significantly up-regulated, with a 2- to 8-fold increase in somatic embryos overexpressing *HSFA9* (Fig. 5B). A significant increase in gene expression level was also observed for two sHSP in somatic embryos overexpressing *DREB2G*. However, overexpression of *HSFA9* or *DREB2G* and downstream sHSP genes was not sufficient to increase the DT of coffee somatic embryos, since there was no statistical difference in survival after equilibrium drying between the HSFA9^+^ or DREB2G^+^ somatic embryos and the controls (Fig. 5C).

## Discussion

Using coffee seeds as a model, we searched for developmental processes that confer desiccation tolerance (DT) in orthodox seeds but that are lacking in intermediate seeds. We showed that DT acquisition occurs late in coffee seed development, i.e. at the end of seed filling, while these events may overlap in seeds whose development is very short, such as Arabidopsis seeds (Leprince *et al.*, 2017 for a review). As in all fleshy fruits, coffee seeds are not subjected to dry ambient air during maturation. DT is therefore acquired without dehydration *in planta* in developing coffee seeds. The programme that governs DT acquisition therefore does not appear to require an environmental cue to start, but appears to be strictly under developmental control. Transcriptome analysis of developing coffee seeds revealed the existence of a major transcriptional switch coinciding with DT acquisition. The period that we define as the late maturation stage in coffee seeds starts with this transcriptional switch at the stage 5–6 transition. It is characterised by the transcriptional activation of several stress-defence mechanisms (antioxidants, detoxication and repair systems, LEAs, HSPs, cell-wall reorganisation) and down-regulation of genes involved in energy processes, primary metabolism, and protein fate. Transcriptome analyses of developing *M. truncatula* and Arabidopsis seeds showed that the same activation and repression processes occur during the late maturation phase of orthodox seeds (see Leprince and Buitink, 2010 for a review). Moreover, none of the DT mechanisms inferred from orthodox seed transcriptomes appeared to be missing in maturing coffee seeds. This observation led us to scrutinise the role played by hormones, LEAs, sHSPs, and TFs thought to play a role in orthodox seed DT.

As in orthodox seeds, ABA appears to play a major role in controlling the late maturation programme in the coffee seed. First, almost all enzymatic steps in ABA biosynthesis were significantly upregulated during DT acquisition. Second, the TF (Cc11_g12740) that displayed the highest up-regulation during DT acquisition (45-fold change) showed very high sequence homology with the Arabidopsis gene *AtANAC029* (NAC III subfamily), which is required for normal Arabidopsis seed development and morphology (Kunieda *et al.*, 2008) and was recently shown to control ABA biosynthesis, notably through trans-activation of the promoter of the abscisic aldehyde oxidase *AAO3* gene (Yang *et al.*, 2014). Our results also suggest the existence of crosstalk between ABA and cytokinins during late maturation. DT acquisition coincided with a marked decrease in cytokinin levels and down-regulation of the cytokinin biosynthetic genes *IPT1* and *LOG1*. Antagonist actions of cytokinins and ABA have already been demonstrated in several biological processes, including seed germination (Guan *et al.*, 2014) and plant survival under abiotic salt and drought stresses (Nishiyama *et al.*, 2011).

Several other transcripts that play a key role in ABA homeostasis and signalling pathways strongly support a role for ABA in coffee seed DT acquisition. ABA 8′-hydroxylase (Cc08_g05920), which is involved in the main ABA catabolic route and regulates ABA levels in mature Arabidopsis seeds (Okamoto *et al.*, 2006), was significantly up-regulated during the stage 5–6 transition. Key components of the ABA sensing (PYR1) and signalling pathways (PP2Cs and SRKI2) also showed very high transcript accumulation during DT acquisition. They included the E3 ubiquitin-protein ligase RGLG1 (Cc07_g18890), which functions as a positive regulator of ABA signalling in Arabidopsis through ABA-dependent degradation of PP2CA (Wu *et al.*, 2016). PP2Cs have been shown to co-ordinate both ABA signalling and the SnRK1-mediated energy-sensing response (Rodrigues *et al.*, 2013). SnRK1, a sensor of energy status that mediates extensive metabolic and developmental reprogramming in response to environmental stresses (Baena-González and Hanson, 2017), is also involved in the co-ordination of metabolic, hormonal, and developmental processes during seed development (Radchuk *et al.*, 2010). SnRK1 is a heterotrimeric protein kinase complex composed of a catalytic subunit, α, and two regulatory subunits, β and γ. In our coffee seed transcriptome, gene expression for the SnRK1 subunit α (*KIN10*, Cc06_g20520) peaked at stage 5, together with *FUS3* with which it has been reported to interact physically to regulate transitions between developmental stages in Arabidopsis (Tsai and Gazzarrini, 2012), while genes for the regulatory subunits KINB1 and KING1 were specifically up-regulated during coffee seed DT acquisition. It is worth noting that silencing of SnRK1β (SNF4) impairs pollen hydration mechanisms in Arabidopsis through modulation of ROS signalling and mitochondrial biogenesis (Gao *et al.*, 2016) and alters seed longevity in *M. truncatula* (Rosnoblet *et al.*, 2007).

It should, however, be noted that the changes in ABA content measured during seed maturation did not reflect transcript accumulation of ABA biosynthetic genes at the stage 5–6 transition: the maximum ABA content was in fact observed at stage 4. It is therefore very likely that most ABA detected during seed filling (stages 4 and 5) was translocated from maternal tissues and then masked local biosynthesis during DT acquisition. This mechanism has been observed in many other plants (Frey *et al.*, 2004; Kanno *et al.*, 2010). The question as to whether translocated ABA and ABA synthesised locally play the same roles in late maturation induction requires further investigation. For instance, in many orthodox seed species, ABA produced by maternal tissues is not sufficient to induce dormancy, which strictly depends on ABA synthesised in the endosperm and the embryo (Kucera *et al.*, 2005).

Several *in vitro* studies have shown that LEAs are intrinsically disordered proteins that can fold reversibly upon desiccation (Tolleter *et al.*, 2007; Hundertmark *et al.*, 2012), and either stabilise membranes (Tolleter *et al.*, 2010; Popova *et al.*, 2011) or prevent protein misfolding and aggregation (Pouchkina-Stantcheva *et al.*, 2007; Boucher *et al.*, 2010; Dang *et al.*, 2014). We identified 29 LEA proteins in the coffee genome. Our transcriptome and proteome analyses revealed that 19 of them were massively transcribed and 17 were translated during coffee seed maturation, and 15 of them were seed-specific. The closest Arabidopsis homologue of each of this subset of 19 LEA genes is also highly transcribed in the maturing Arabidopsis seed (Hundertmark and Hincha, 2008; also visualised with the eFP browser by Winter *et al.*, 2007; Supplementary Table 1Supplementary Table S7). Within this maturation subset, 13 LEA genes were significantly up-regulated during DT acquisition. The closest Arabidopsis homologues of nine LEAs of this coffee seed DT subset are associated with DT re-induction in pre-germinated Arabidopsis seeds (Supplementary Table 1Supplementary Table S7; Maia *et al.*, 2011) and three of them also display high homology with one of the LEAs involved in DT re-induction in *M. truncatula* pre-germinated seeds (Supplementary Table 1Supplementary Table S7; Boudet *et al.*, 2006). Also within this DT subset, we found a LEA homologous to EM6 (73% identity at the amino acid level), which plays a critical role in water binding during Arabidopsis seed maturation (Manfre *et al.*, 2009). In conclusion, our focus on LEA proteins did not reveal a specific lack in coffee seeds in comparison with orthodox seeds. Although accumulation of LEAs is a process shared by most anhydrobiotes, and ectopic expression of plant LEA genes may enhance DT in yeast (Dang *et al.*, 2014), the specific role of LEAs in seed DT acquisition remains to be revealed. The timing of accumulation of most LEAs coincides with longevity rather than with DT acquisition during orthodox seed development (Chatelain *et al.*, 2012; Leprince *et al.*, 2017). Several Arabidopsis mutants that lack one or two LEA proteins are still desiccation tolerant (Carles *et al.*, 2002; Hundertmark *et al.*, 2011). Silencing of dehydrins alters Arabidopsis seed longevity under controlled deterioration without affecting DT (Hundertmark *et al.*, 2011). Similarly, *Castanospermum australe* seeds accumulate large amounts of dehydrins but are recalcitrant (Delahaie *et al.*, 2013). How LEAs contribute to seed DT thus requires further clarification.

sHSPs are ATP-independent chaperones that prevent protein aggregation by maintaining them in a folding-competent state and that refold misfolded proteins alone or in concert with other ATP-dependent chaperones. Several seed-specific heat-shock factors, including HSFA9 (Kotak *et al.*, 2007), are responsible for HSP transcriptional activation and accumulation during late maturation in Arabidopsis (Wehmeyer *et al.*, 1996). The hypothesis that sHSPs contribute to seed DT has been supported by enhanced DT in desiccation-sensitive vegetative organs ectopically expressing HSFs (Prieto-Dapena *et al.*, 2008; Personat *et al.*, 2014). In the present study, we also demonstrated increased sHSP transcript accumulation in coffee somatic embryos overexpressing *HSFA9* or *DREB2*. By contrast, *HSFA9* or *DREB2* overexpression did not confer desiccation tolerance to transformed embryos. In common with LEA proteins, while several studies suggest that sHSPs play a major role in seed longevity (Prieto-Dapena *et al.*, 2006; Almoguera *et al.*, 2009; Kaur *et al.*, 2015), their contribution to seed DT requires further investigation.

In conclusion, the present work revealed that the cellular and regulation processes that occur during late maturation in the coffee seed are remarkably similar to those known or thought to be involved in orthodox seed DT. Two hypotheses, which are not mutually exclusive, thus deserve to be explored to understand the impaired tolerance to drying of intermediate seeds. First, the differences are quantitative. For instance, the metabolic shutdown may be only partial in intermediate seeds whereas it is hypothesised to be complete in orthodox seeds, or the amount of a certain type of LEA accumulated in intermediate seeds may be smaller than in orthodox seeds. Second, intermediate seeds may have certain particularities that are not modified by transcriptional changes during DT acquisition. For instance, a peculiar membrane lipid composition of these tropical seeds could result in specific lethal phospholipid phase transitions at low RH that would not be prevented by membrane stabilisers up-regulated during DT acquisition. The interspecific variability of seed DT that exists within the genus *Coffea* (Dussert *et al.*, 1999) could be very useful to test these two hypotheses.

## Supplementary data

Supplementary data are available at *JXB* online.

Table S1. List of specific primers used for real-time RT-PCR and PCR.

Table S2. Proteins detected in the heat-stable proteome of the mature coffee seed.

Table S3. Phytohormone profiling in developing coffee seed: ESI-MS-MS analysis of ABA, auxin, cytokinin, and gibberellin families.

Table S4. Relative expression of the 15 522 genes represented on the oligonucleotide PUCE CAFE microarray.

Table S5. Gene composition of differentially down- (cluster C5) and up-regulated genes (clusters C18–C26) during DT acquisition.

Table S6. GO and MapMan enrichment analyses of clusters of down-regulated genes (cluster C5) and up-regulated genes (clusters C18–C26) during DT acquisition.

Table S7. Genome-wide analysis of the coffee LEA gene family.

Table S8. Endosperm and embryo transcriptomes (454) during the stage 5–6 transition.

Fig. S1. Hierarchical cluster analysis of the 5857 genes displaying significant up- or down-regulation at one of the six developmental transitions.

Fig. S2. Phylogenetic tree of the 29 coffee and 51 *Arabidopsis thaliana* LEA proteins.

Fig. S3. Comparisons of the amino acid sequence of HSFA9 proteins from *Helianthus annuus* (AAM43804) and coffee (Cc06_g11570), and of the amino acid sequence of DREB2G proteins from *Arabidopsis thaliana* (At5g18450) and coffee (Cc09_g03140).

Fig. S4. PCR detection of the HPTII hygromycin-resistance gene in transgenic coffee plants.

Supplementary_Figures_S1_S4

supplementary_tables_S1_S8
