# Supplementary material for: Integrative analysis of the late maturation programme and desiccation tolerance mechanisms in intermediate coffee seeds
Source: J Exp Bot. 2018 Jan 29;69(7):1583–97. doi: 10.1093/jxb/erx492 (PMC5888931; doi:10.1093/jxb/erx492)
Supplement: Supplementary_Figures_S1_S4 [file erx492_suppl_supplementary_figures_s1_s4.pdf]

Integrative analysis of the late maturation programme and desiccation tolerance mechanisms in intermediate coffee seeds

Stéphane Dussert, Julien Serret, Aldecinei Bastos-Siqueira, Fabienne Morcillo, Eveline Déchamp, Valérie Rofidal, Philippe Lashermes, Hervé Etienne, Thierry Joët

Supplemental Figures S1-S4.

Figure S1. Hierarchical cluster analysis of the 5 857 genes displaying significant up- or down-regulation at one of the six developmental transitions. Hierarchical clustering analysis grouped 1 521 genes (26%, 9 clusters) which were specifically up-regulated during DT acquisition: i.e. genes that were significantly up regulated only at the stage 5-6 transition. Similarly, 613 genes (13%) were found to be expressed throughout endosperm growth and filling but specifically repressed during the late maturation phase.

Figure S2. Phylogenetic of the 29 coffee and 51 Arabidopsis thaliana LEA proteins. Phylogenetic analyses were performed on the Phylogeny.fr platform (http://www.phylogeny.fr). Sequence alignment was performed using the ClustalW algorithm and the dendrogram was constructed using the neighbour-joining method. Full-length deduced amino acid sequences were used for distance analysis. Numbers on the branches are bootstrap values for 1 000 replicates. The different LEA groups (as defined by Hundertmark and Hincha, 2008) are indicated by different colours: yellow (LEA_1), pink (LEA_2), orange (LEA_3), blue (LEA_4), brown (LEA_5), green (dehydrins) and grey (seed maturation proteins, SMP).

Figure S3. A. Comparison of the amino acid sequence of HSFA9 proteins from Helianthus annuus (AAM43804) and coffee (Cc06_g11570). Sequence alignment of the longest translated open reading frames was performed using Clustal W: 47.1% identity in 367 amino acid overlap. Ha, Helianthus annuus, Ca, Coffea arabica. Functional modules are indicated by an open box: DBD, DNA-binding domain; HR-A/B, hydrophobic repeat A/B; NLS, nuclear localization signal; AHA, aromatic and hydrophobic amino acid residues embedded in an acidic environment. For further description of the modules, see Nover et al., 2001 (Cell Stress & Chaperones 6, 177-189). B. Comparison of the amino acid sequence of DREB2G proteins from Arabidopsis thaliana (At5g18450) and coffee (Cc09_g03140). Sequence alignment of the longest translated open reading frames was achieved using Clustal W: 57% identity in 207 amino acid overlap (74.9% similarity).

Figure S4. PCR detection of the HPTII hygromycin resistance gene in transgenic coffee plants. Somatic embryos and regenerated plantlets were produced from 6-month-old embryogenic callus cultures that were co-cultivated for five days with A. tumefaciens strain LBA1119 with the pMDC32 binary vector, either empty or containing the coffee HSFA9 or DREB2 gene, and then cultivated for five months on a decontamination medium containing decreasing amounts of cefotaxime (1000mg/L, 500mg/L, 250mg/L). M - Molecular weight DNA markers (1 kb), C - untransformed coffee plant (control), 1-12 transgenic coffee plants derived from independent transformation events, B - blank, PCR mix without DNA, P - plasmid. The arrow indicates the fragment corresponding to the HPTII gene (506 bp).

Supplementary Figure S1

Hierarchical cluster analysis (HCA) of the 5857 genes displaying significant up- or down-regulation at one of the six developmental transitions. For each of the 26 major clusters revealed, the number of belonging genes is given in brackets.

Hierarchical clustering analysis grouped 1521 genes (26%, 9 clusters C18 to C26) which were specifically up-regulated during DT acquisition: i.e. genes that were significantly up regulated only at the stage 5-6 transition. Similarly, 613 genes (13%, Cluster C5) were found to be expressed throughout endosperm growth and filling but specifically repressed during the late maturation phase.

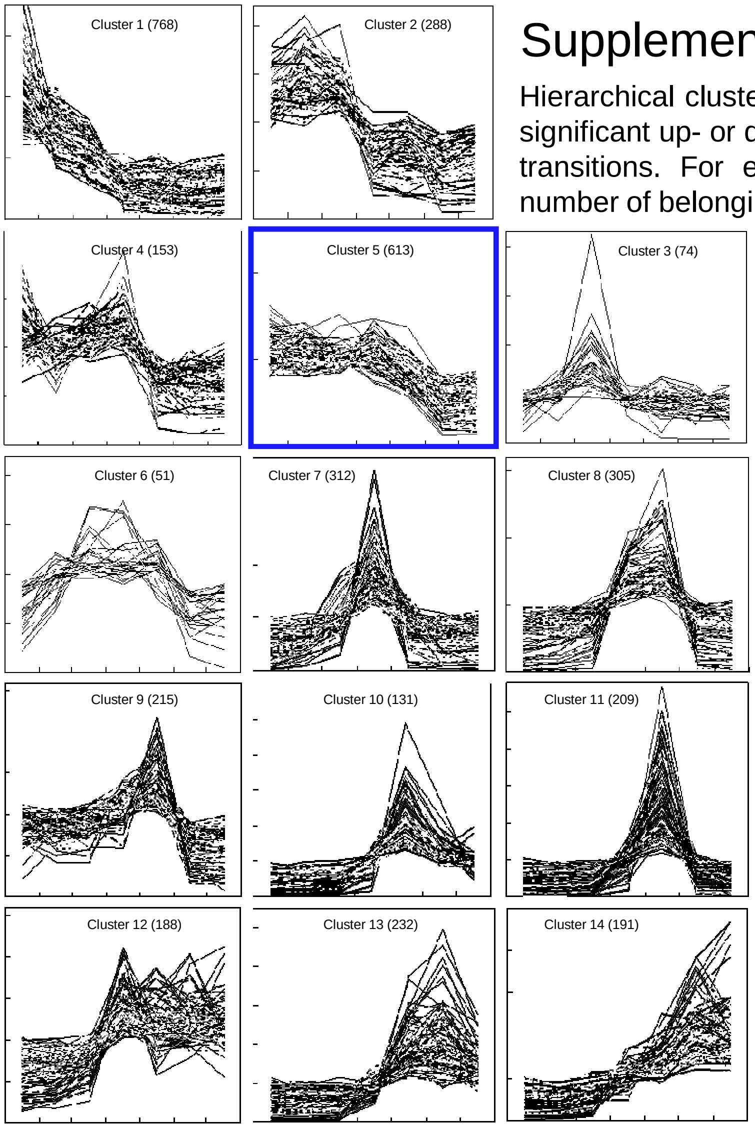

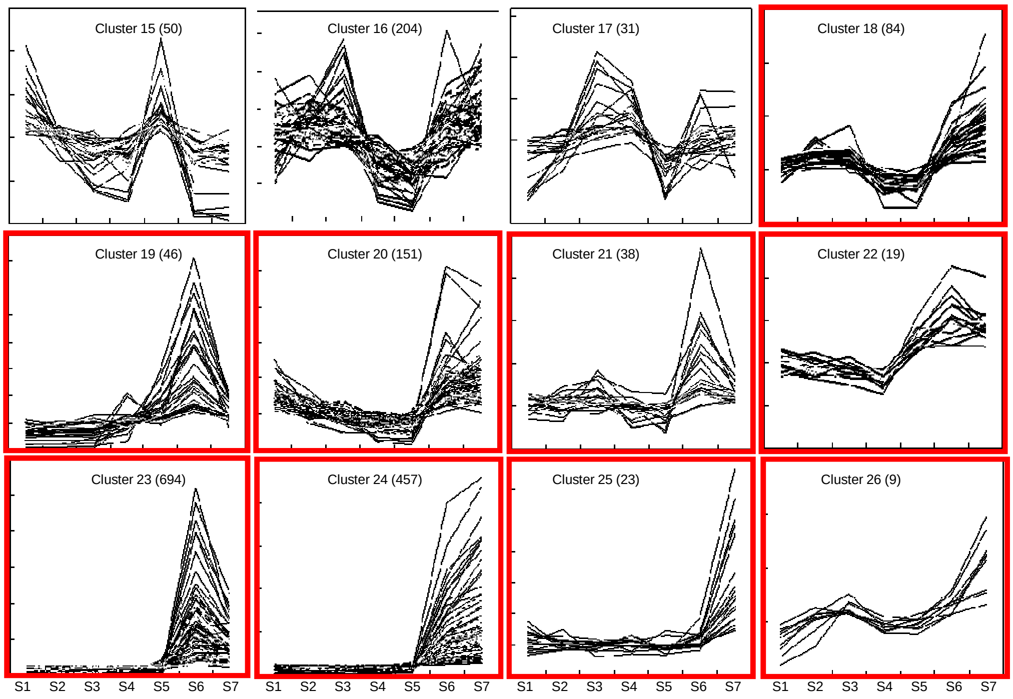

Supplementary Figure S2

Phylogenetic of the 29 coffee and 51 *Arabidopsis thaliana* LEA proteins. Phylogenetic analyses were performed on the Phylogeny.fr platform (http://www.phylogeny.fr). Sequence alignment was performed using the ClustalW algorithm and the dendrogram was constructed using the neighbour-joining method. Full-length deduced amino acid sequences were used for distance analysis. Numbers on the branches are bootstrap values for 1 000 replicates. The different LEA groups (as defined by Hundertmark and Hincha, 2008) are indicated by different colours: yellow (LEA_1), pink (LEA_2), orange (LEA_3), blue (LEA_4), brown (LEA_5), green (dehydrins) and grey (seed maturation proteins, SMP).

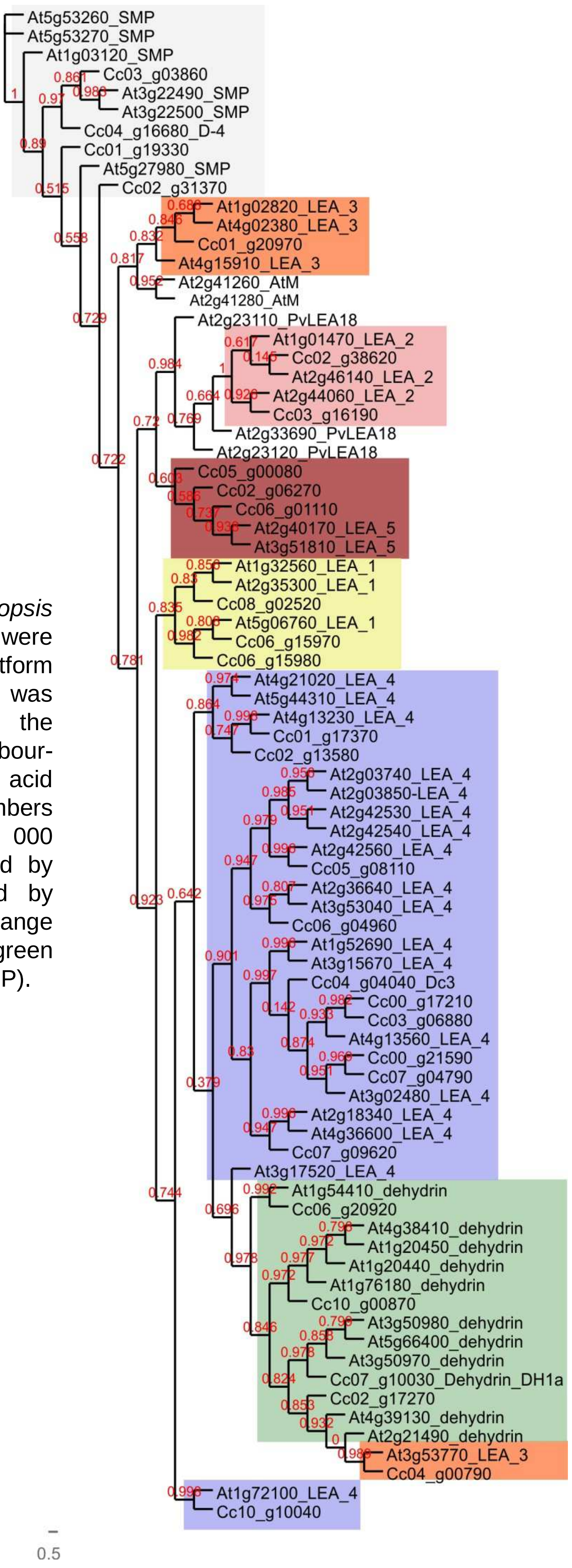

A

```
                                                           DBD
HaHSFA9 33  EMMKIKEEPMLVFDEH-------EDFVGGEE---APKPIEGLRDGGPPPFLK[illegible]FEMVDD
CaHSFA9 26  EILTVKEEPVVFLDEDGPFGNVSSNFSGGGEFSDVPKPLQGLHEVGPPPFLKKTFEMVDD
            :.. .::::.. .::         .: :: :   .:::..::.. :::::::::::::::
             DBD
HaHSFA9 83  PTTDSIISWSSSKNSFILWDPHKFSTDLLPQRFKHNNFSSFVRQLNTYRFKKIDPDRWEF
CaHSFA9 86  PETDSTISWSSTNTSFVVWDPHKFSRDLLPKHFKHNNFSSFVRQLNTYRFRKTDSDRWEF
            : ::: :::::...::..::::::: ::::..::::::::::::::::::.: : :::::
             DBD                                                HR-A
HaHSFA9 143 ANEFFQKGKKHLLRDIKRRTNQPQNTQKQEEIRKQEQQQCCGHQTNSTMETELKNLRKER
CaHSFA9 146 ANEEFQKGKKHLLKNIKRRKQHSQMLQHQGAGQPWLDS------ANYISETELQKLRNDQ
            ::: :::::::::..:::: .. :  :.:   .   ..      .:   ::::..::...
             HR-A                                  HR-B
HaHSFA9 203 ITLKQEILKMKQQQENTEKHLEMVEERMLRMEFKQQQLLVFMSKAFRNPIFVKL-LQHLV
CaHSFA9 200 NTLKLELLRLKQQQVNTENYLAAVKERLRTAESKQKYMAIFIVKAFKNPLFVQLFIEKMK
             ::: :.:..:::: :::..:  :.::.   : ::. . .:. :::.::.::.: ....
             NLS                           AHA1
HaHSFA9 262 QKQKTGSVEMCKKRKLEQMLNTDDLDRFQEMWNM-------------IEP-DAYTVLSSD
CaHSFA9 260 QKRALGSGEVSKKRRLAGPQGNENLTEAMNAANNSLDATRKAVDGKNLQPQDEVTTVDPE
            ::.  :: :. :::.:    ....: . ..  :              ..: :  :... .
                                          AHA2
HaHSFA9 308 GSVSPPEDQNTS---DKSGSNGSDYNSESFILWEKLMEDELIFGGE-QSGKDQTETYLQE
CaHSFA9 320 IQILFSPDHESSGPLQEQLVGASSNTSENFILWEKLMEDDMIYENEPETGKSQSEIVL-E
             ..    :...:   ...  ..:.  .::.::::::::::..:.:. ..::.:.:  :  :
             AHA3
HaHSFA9 364 WEELIPK
CaHSFA9 379 LESLIAK
             : :: :
```

B

```
AtDREB2G 1   SPPDREGARATHDEHYDRATI-NRESP-NSIVEELEMENTBINDINGPR-TEINGSARAB
                    :  ..::::::::: ::::: ::::::::::::::::::::: ::::     .
CaDREB2G 1    CCGPCTATIVEDEHYDRATIKNRESPKNSIVEELEMENTBINDINGPRKTEIN-----D


AtDREB2G 57  IDPSISTHALIANAGNDREBGPESVMEEEQPPAKKRNMGRSRKGCMKGKGGPENATCTFR
             .   . .... ..   :..   :...   .   :: ..: ::::::.:::::::: ::.:
CaDREB2G 55  MMERVENEGVNVGIPVDKKPKKEAAI---STSMKKITVGGSRKGCMRGKGGPENAFCTYR


AtDREB2G 118 GVRQRTWGKWVAEIREPNRGTRLWLGTFNTSVEAAMAYDEAAKKLYGHEAKLNLVHPQQQ
             ::::::::::::::::::.:.:.:::::::: ::: :::.:::.:::. :::::  :...
CaDREB2G 112 GVRQRTWGKWVAEIREPNHGARVWLGTFNTSYEAARAYDDAAKRLYGKCAKLNL--PEED


AtDREB2G 177 QQVVVNRNLSFSGHGS-GSWAYNKKLDMVHGLDLGLGQASCSRGSCSERSSFLQEDDDHS
             :        :  : .: .: ::...  . .: ::
CaDREB2G 170 QPP------SPPGSSSVASAAYSNNTGYKNGQDLTNEHQSPELEDKKNGTSVLDEVSIFK


AtDREB2G 237 HNRCSSSSGSNLCWLLPKQSDSQDQETVNATTSYGGEGGGGSTLTFSTNLKPKNLMSQNY

CaDREB2G 224 DINGEFAFDETPAPSLLGEEQILNWPEYPFDNGFHWSNDGGISVGGLIDHAVVYKLLGPP
```

Supplementary Figure S3

A. Comparison of the amino acid sequence of HSFA9 proteins from *Helianthus annuus* (AAM43804) and coffee (Cc06_g11570). Sequence alignment of the longest translated open reading frames was performed using Clustal W: 47.1% identity in 367 amino acid overlap. Ha, *Helianthus annuus*, Ca, *Coffea arabica*. Functional modules are indicated by an open box: DBD, DNA-binding domain; HR-A/B, hydrophobic repeat A/B; NLS, nuclear localization signal; AHA, aromatic and hydrophobic amino acid residues embedded in an acidic environment. For further description of the modules, see Nover *et al.*, 2001 (Cell Stress & Chaperones 6, 177-189) . **B.** Comparison of the amino acid sequence of DREB2G proteins from *Arabidopsis thaliana* (At5g18450) and coffee (Cc09_g03140). Sequence alignment of the longest translated open reading frames was achieved using Clustal W: 57% identity in 207 amino acid overlap (74.9% similarity).

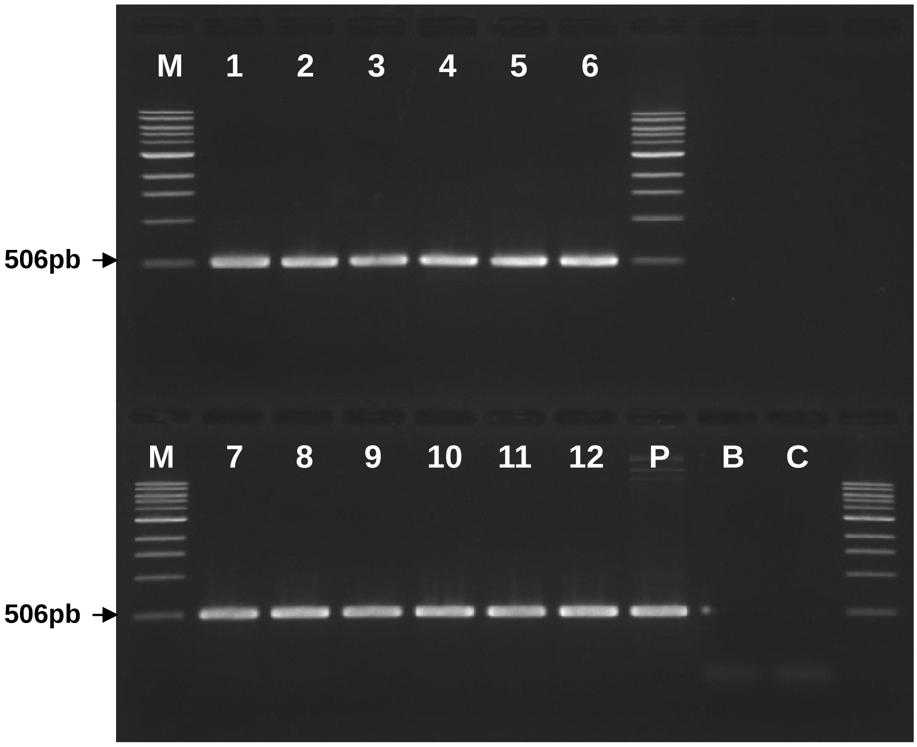

cDNAs derived from somatic embryos:

1: HSFA9+ a
2: HSFA9+ b
3: HSFA9+ c
4: HSFA9+ d
5: DREB2+ a
6: DREB2+ b
7: DREB2+ c
8: DREB2+ d
9: pMDC32 empty a
10: pMDC32 empty b
11: pMDC32 empty c
12: pMDC32 empty d
P: pMDC32 plasmid (control)
B: Blank, PCR mix without DNA
C: Control, untransformed coffee embryo
M: 1kb Molecular weight DNA markers

Supplementary Figure S4

PCR detection of the HPTII hygromycin resistance gene in transgenic coffee plants. Somatic embryos and regenerated plantlets were produced from 6-month-old embryogenic callus cultures that were co-cultivated for five days with *A. tumefaciens* strain LBA1119 with the pMDC32 binary vector, either empty or containing the coffee *HSFA9* or *DREB2* gene, and then cultivated for five months on a decontamination medium containing decreasing amounts of cefotaxime (1000mg/L, 500mg/L, 250mg/L). M - Molecular weight DNA markers (1 kb), C - untransformed coffee plant (control), 1-12 transgenic coffee plants derived from independent transformation events, B - blank, PCR mix without DNA, P - plasmid. The arrow indicates the fragment corresponding to the HPTII gene (506 bp).
